# Magnitude, Associated Risk Factors, and Trend Comparisons of Urinary Tract Infection among Pregnant Women and Diabetic Patients: A Systematic Review and Meta-Analysis

**DOI:** 10.1155/2023/8365867

**Published:** 2023-07-28

**Authors:** Abayeneh Girma, Aleka Aemiro, Dereba Workineh, Dessalew Tamir

**Affiliations:** ^1^Department of Biology, College of Natural and Computational Science, Mekdela Amba University, P.O. Box 32, Tulu Awlia, Ethiopia; ^2^Department of Biology, College of Natural and Computational Science, Mizan-Tepi University, P.O. Box 121, Tepi, Ethiopia; ^3^Department of Veterinary Science, College of Agriculture and Environmental Sciences, Debre Tabor University, P.O. Box 272, Debre Tabor, Ethiopia

## Abstract

Urinary tract infection (UTI) remains the most common bacterial infection that affects millions of people around the world, especially pregnant women (PW) and people with diabetes mellitus (DM). This systematic review and meta-analysis was aimed at finding the pooled prevalence of UTI and its associated risk factors among PW and DM patients. Scientific articles written in English were recovered from PubMed, ScienceDirect, Web of Science, Google Scholar, Cochrane Library, Google Engine, and University Library Databases. “Prevalence,” “urinary tract infection,” “associated factors,” “pregnant women,” “diabetic patients,” and “Ethiopia” were search terms used for this study. For critical appraisal, PRISMA-2009 was applied. Heterogeneity and publication bias were evaluated using Cochran's *Q*, inverse variance (*I*^2^), and funnel plot asymmetry tests. A random effect model was used to calculate the pooled prevalence of UTI and its associated factors among both patients, along with the parallel odds ratio (OR) and 95% confidence interval (CI). For this meta-analysis, a total of 7271 participants were included in the 25 eligible studies. The pooled prevalence of UTI in Ethiopia among both patients was 14.50% (95% CI: 13.02, 15.97), of which 14.21% (95% CI: 12.18, 16.25) and 14.75% (95% CI: 12.58, 16.92) were cases of DM and PW, respectively. According to the subgroup analysis, the highest prevalence was observed in the Oromia region (19.84%) and in studies conducted from 2018 to 2022 (14.68%). Being female (AOR: 0.88, and 95% CI: 0.11, 1.65, *P* = 0.01) and having an income level ≤ 500ETB (AOR: 4.46, and 95% CI: -1.19, 10.12, *P* = 0.03) were risk factors significantly associated with UTI among patients with DM and PW, respectively. Furthermore, a history of catheterization (AOR = 5.58 and 95% CI: 1.35, 9.81, *P* < 0.01), urinary tract infection (AOR: 3.52, and 95% CI: 1.96, 5.08, *P* < 0.01), and symptomatic patients (AOR: 2.32, and 95% CI: 0.57, 4.06, *P* < 0.01) were significantly associated with UTI in both patients. Early diagnosis and appropriate medication are necessary for the treatment of UTI in patients with DM and PW.

## 1. Introduction

Urinary tract infection (UTI) is defined as the colonisation of a pathogen in any part of the urinary tract, including the kidney, ureter, bladder, and urethra. Infection of the urinary tract is one of the most common infectious diseases, affecting people of all ages and causing about 150 million global cases per year, in addition to costing the global economy over $6 billion in treatment costs [1, 2]. UTIs are classified according to their location of infection (pyelonephritis (kidney), cystitis (bladder), and urethritis (urethra)) as well as their severity (complicated versus uncomplicated) [3]. Uncomplicated UTIs affect people who are otherwise healthy and do not have anatomical or neurological problems with their urinary system. These infections are classified as lower UTIs (cystitis) or higher UTIs (urethritis) or pyelonephritis. Cystitis can be caused by a number of factors, including current symptoms of UTI, a history of UTI and catheterisation, sexual activity, vaginal infection, diabetes, obesity, and genetic predisposition [4]. Complicated UTIs are those that are linked to factors that influence the urinary tract or the host's immune system. Urinary blockage, urine retention owing to neurological disease, immunosuppression, renal failure, renal transplantation, pregnancy, and the presence of foreign substances such as calculi, indwelling catheters, or other drainage devices are all potential complications of UTIs [5].

Diabetes mellitus (DM) is one of the most common noncommunicable diseases, affecting the health of a large proportion of the global population. The presence of fasting blood glucose levels greater than 126 mg/dL is a key symptom of diabetes mellitus [6]. Globally, the prevalence is expected to increase from 171 million in 2000 to 366 million by 2030 [7]. The frequency of diabetes mellitus is rising across Africa, and the disease's severity is worsening [8]. More than 12 million people in sub-Saharan Africa are predicted to have diabetes mellitus, with 330,000 of them dying from its complications [9]. According to WHO estimates, the number of diabetic cases in Ethiopia was 800,000 in 2000, and this figure is expected to increase to 1.8 million by 2030 [10]. Although diabetes mellitus is considered one of the most serious noncommunicable illnesses in Ethiopia, its exact prevalence, progression, and complications are not adequately documented or updated on a regular basis.

Urinary tract infections are associated with considerable morbidity in both the mother and the baby during pregnancy. The combination of mechanical, hormonal, and physiological changes that occur during pregnancy causes significant changes in the urinary system that have a considerable impact on the acquisition of bacteriuria and its natural history [11]. Infections of the urinary tract during pregnancy can result in poor pregnancy outcomes and complications such as preterm delivery, low birth weight, preeclampsia (toxaemia), and anaemia. Therefore, it is important to check and treated as soon as possible. Prenatal screening is not considered a necessary aspect of antenatal care in most impoverished countries, including Ethiopia [12].

The identification of the types of organisms that cause urinary tract infections in diabetes mellitus (DM) and pregnant women (PW) patients, as well as the selection of an effective antibiotic against the organism in question, is critical to the successful care of these individuals. The rise of resistant bacterial strains in hospitals continues to represent a problem in terms of the treatment and control of disease transmission. Furthermore, the indiscriminate use of antibiotics often leads to an increase in resistant urinary pathogens to the most commonly used antimicrobial medications, especially in patients with diabetes and pregnant women. Although UTIs rarely cause complications, they can have serious consequences in terms of morbidity and mortality [13]. According to various studies, the prevalence of UTI is increasing in Ethiopia. In a few hospital-based studies conducted in Ethiopia's central and northwest regions, rates of antibiotic resistance in urinary tract infections ranged from 10.4% to 17.8%, with a greater rate of multidrug resistance in diabetic patients ranging from 59.8% to 71.7 percent [14]. In Ethiopia, the prevalence of urinary tract infections (UTIs) among pregnant women varies greatly; it ranges from 9.8% to 26.6% [15]. In addition, the isolates were found to have a significant level of resistance to routinely used antibiotics, leaving clinicians with a limited number of options for treating UTIs.

Sex, illiteracy, history of catheterization, blood glucose level, type of diabetes, duration of DM, insulin therapy, cigarette smoking, and history of UTI have been identified as important risk factors for UTI among diabetes patients [16], while sociodemographic factors such as maternal age, residence, marital status, maternal educational status, monthly family income, and maternal occupation, as well as medical and obstetric-related factors such as anaemia, HIV status, history of UTI, history of catheterization, parity, and gestational age, have been identified as potential-associated factors for UTI in pregnant women [15, 17].

Urinary tract infections are one of the most common public health problems, with varying levels of prevalence throughout the country. Even in Ethiopia, the prevalence of UTI and its predisposing factors are not well collected, organised, or recorded as a systematic review and meta-analysis. As a result, the objective of this study was to provide evidence on the general prevalence and risk factors for UTIs among patients with DM and PW using previously conducted research articles. Furthermore, the results obtained in the current investigation could significantly benefit healthcare providers, users, and policymakers.

## 2. Methods

### 2.1. Country Profile

Ethiopia measures 1,104,300 square kilometers and is located in the Horn of Africa. The total land area is 1,000,000 square kilometers (386,102 square miles). Ethiopia is bordered to the north by Eritrea, to the east by Djibouti and Somalia, to the west by Sudan and South Sudan, and to the south by Kenya. According to Worldometer's elaboration of the most recent United Nations data, Ethiopia's current population was 113,881,451 in 2020, which is comparable to 1.47 percent. Furthermore, the aforementioned report predicts that by 2020, approximately 21.3% of the population (24,463,423) will live in urban areas [18, 19].

### 2.2. Search Strategy

**Figure 1:**
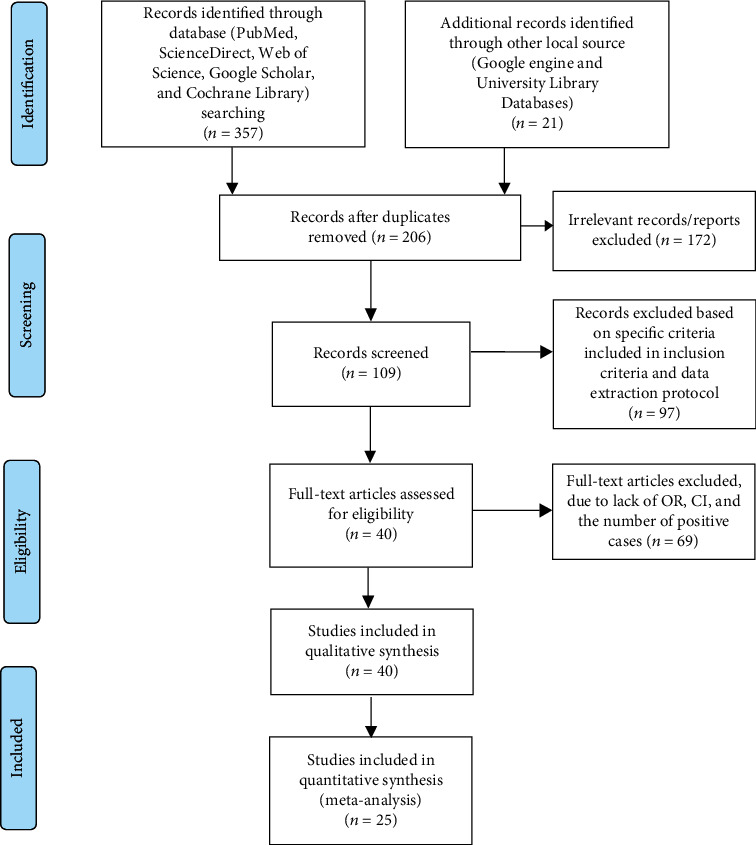
PRISMA-2009 flow diagram of eligible studies.

This systematic review and meta-analysis were performed according to the guidelines for preferred reporting items for systematic review and meta-analysis (PRISMA) [20]. An extensive search was conducted in international databases (PubMed, ScienceDirect, Web of Science, Google Scholar, and the Cochrane Library) and other sources (Google Engine and University Library Databases). Journals were searched using key terms and phrases such as “prevalence,” “urinary tract infection,” “pregnant women,” “diabetic patients,” “associated risk factors,” and “Ethiopia”. The study was conducted from November 2021 to June 2022. The search process was presented as per PRISMA-2009 flow chart guidelines that clearly indicate the studies included and excluded, along with reasons for exclusion (Figure 1).

### 2.3. Criteria for the Inclusion and Exclusion of Studies

In this systematic and meta-analytic review, institutional, hospital, and community-based studies were included. Articles collected through the searches were evaluated for inclusion in the meta-analysis based on the following criteria: (i) Ethiopian studies on the prevalence of UTI and their risk factors with at least 160 observations; (ii) only human studies reported in English with clearly stated sample sizes, number of positive samples, and study locations; (iii) cross-sectional studies; (iv) journals studied from 2012 to 2022; (v) articles published and available online; (vi) articles used the culture method for the detection of UTI; (vii) reported both asymptomatic and symptomatic UTIs; and (viii) only studies reported bacterial etiological agents. However, reports on the knowledge and practise of diabetic patients or pregnant women towards UTI, investigate patterns of antimicrobial resistance only, only asymptomatic studies, other etiological agents (fungal and protozoan), duplicate publications or extensions of the analysis of the original studies, and studies that were incompletely presented were excluded from the review process. Among many of the previously published articles, only 25 met the selection criteria of the meta-analysis (Figure 1).

### 2.4. Data Extraction

Microsoft Excel (2010) and STATA version 14 software were used for data extraction and analysis. The data extraction protocol consists of the name of the country, author and year of publication, region, study area, type of UTI patients (pregnant women or diabetics), study setting, study design, sample taken, laboratory method used for detection of UTI, type of UTI (asymptomatic or symptomatic or both), etiological agents (bacterial, fungal, protozoan, or all), sample size, number of positive cases, prevalence of UTIs, quality assessment, and their associated risk factors. If the study was conducted over a range of years, then the latest year of the stated range was used. The period from January 1 to March 30, 2022, was used for study selection, quality evaluation, and data extraction.

### 2.5. Quality Assessment of Individual Studies

The general quality of the evidence was evaluated using the GRADE approach (recommendations assessment, development, and evaluation) [21]. Using the three main assessment tools (methodological quality, comparability, study outcome, and statistical analysis), the quality of each study was determined. High-quality publications received 5 to 6 points, moderate-quality publications received 4 points, and low-quality articles received 0 to 3 points. The choice and evaluation of the articles' quality were done independently by four reviewers (A.G., A.A., D.W., and D.T.). The articles were added after the agreement was reached, and discrepancies between the reviewers were resolved through discussion.

### 2.6. Risk of Publication Bias

Using funnel plot symmetry, Cochran's *Q* test, and the *I*^2^ test, the risks of publication bias and heterogeneity were analysed.

### 2.7. Statistical Analysis

The pooled prevalence of UTIs among patients with DM and PW was calculated by dividing the total number of positive cases by the total number of study subjects included in this meta-analysis and multiplying by a factor of 100. A random effect model was used to estimate the size of the pooled effects. To sort out the causes of heterogeneity, subgroup analysis was conducted based on sample size, region of the study, study setting, type of patients, and the year of publication. The Cochran *Q* statistic with inverse variance (*I*^2^) and funnel plot symmetry was used to assess the existence of statistical heterogeneity. The Cochran *Q* statistic was used to determine whether heterogeneity was present between studies. While the heterogeneity (heterogeneity between studies) was measured using the *I*^2^ statistic, values of 25, 50, and 75%, respectively, indicated moderate, medium, and high heterogeneity [22]. A log odds ratio was used to decide the association between UTIs and associated risk factors among respondents included in the studies. Meta-analysis was performed using Stata software version 14, where *P* < 0.05 was considered statistically significant.

## 3. Results

A total of 378 articles on the prevalence and associated risk factors for UTI among diabetic patients and pregnant women in Ethiopia were retrieved. Of 378 articles, one hundred seventy-two of these articles were excluded due to duplicates. From the remaining 206 articles, 97 were excluded based on specific criteria included in the inclusion criteria and data extraction protocol. Of the remaining 109 articles, 69 were also excluded due to the fact that they did not have OR, 95% CI, or the number of positive cases (which means that the report was based only on the estimated prevalence percentage). Thus, only 40 and 25 of the studies, respectively, met the eligibility criteria and were included in the final systematic review and meta-analysis study (Figure 1).

### 3.1. Characteristics of the Eligible Studies

Table 1 presents the characteristics of the studies eligible for analysis. Forty and twenty-five studies were eligible for systematic reviews (Table 1) and meta-analyses (Table 2), respectively. The studies included in the meta-analysis were conducted between 2012 and 2022, and all were cross-sectional studies. Six studies had ≤200 sample sizes, while 19 articles had >200 samples. Nine and sixteen studies were conducted between 2012 and 2017 and 2018 and 2022, respectively. Regarding the types of patients, 12 articles were included for patients with diabetes, and 14 were for pregnant women. Based on the criteria, Amhara (9 articles), Oromia (5 articles), eastern (Harari, Dire Dawa, and Somali) Ethiopia (5 articles), Sidama (4 articles), and Addis Ababa (2 articles) were involved. Nineteen, four, and three articles were carried out in hospital, institutional, and facility settings, respectively. The prevalence of DM among eligible studies ranged between 9.8% and 20.2%. Furthermore, the prevalence of UTI among PW ranged from 7.8% to 26.0% (Table 2).

### 3.2. Pooled Prevalence of UTI

**Figure 2:**
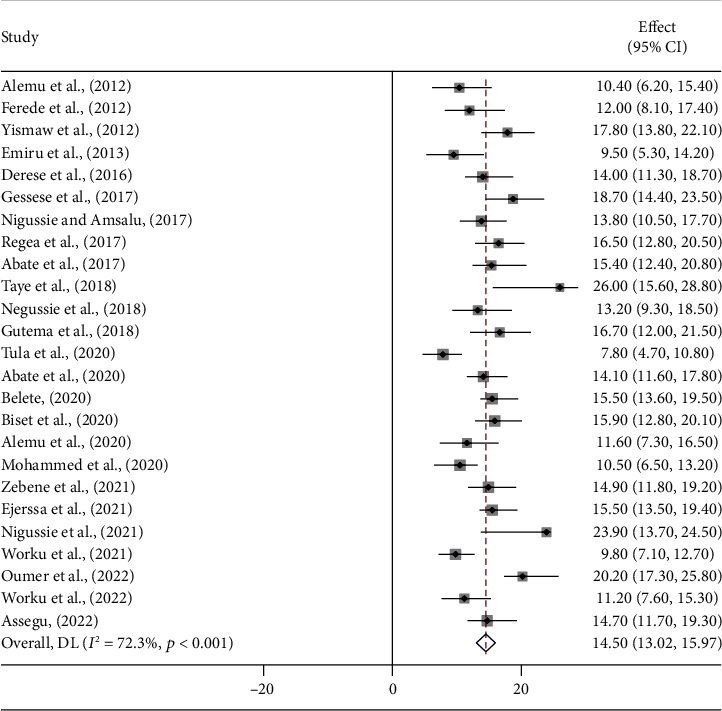
Pooled prevalence forest plot of UTI among DM and PW patients in Ethiopia from 2012 to 2022.

A sensitivity analysis was performed using a random effect model to examine the effects of the individual-included studies on the pooled prevalence of UTI in Ethiopia. The results showed that no single study had an impact on the combined prevalence of UTI among patients with DM and PW. The overall national prevalence of UTI among patients with DM and PW was 14.50 (95% CI: 13.02, 15.97) (Figure 2).

### 3.3. Subgroup Analysis

**Figure 3:**
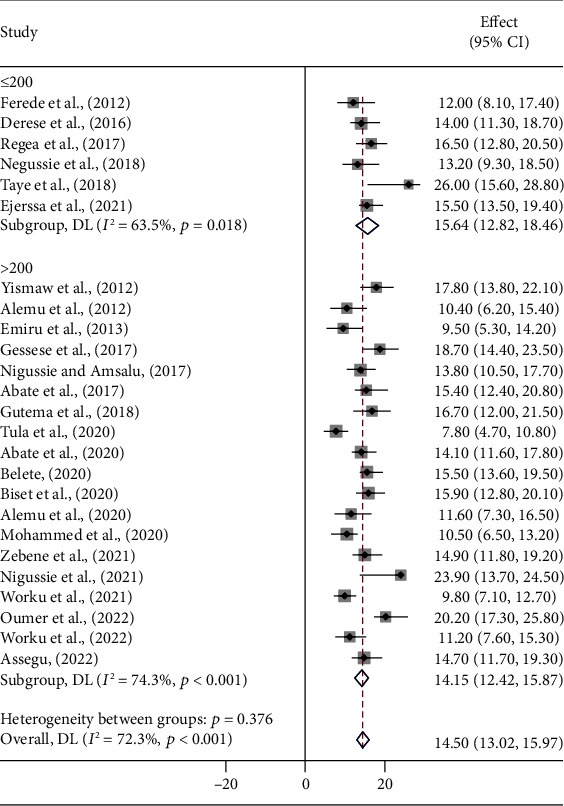
Pooled prevalence of UTIs among patients with DM and PW by sample size.

**Figure 4:**
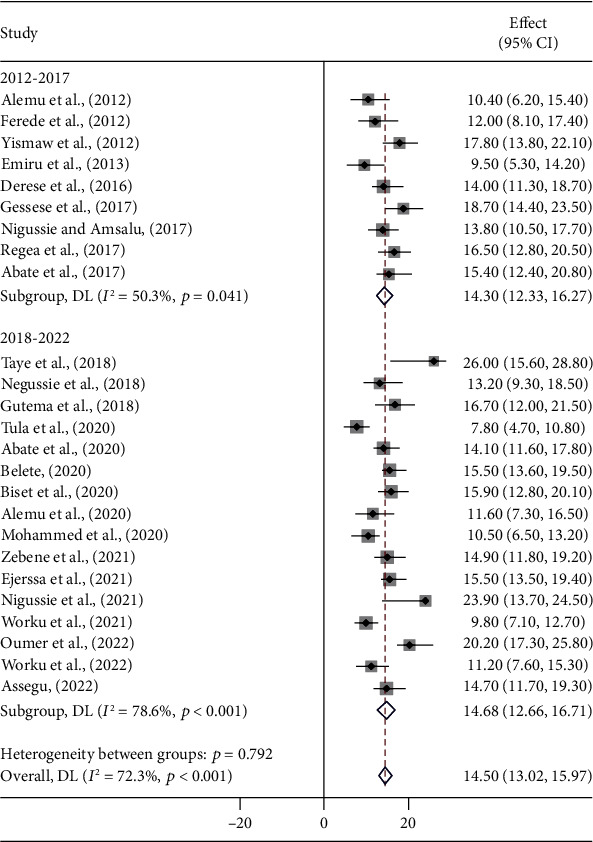
Pooled prevalence of UTI among patients with DM and PW between 2012-2017 and 2018-2022.

**Figure 5:**
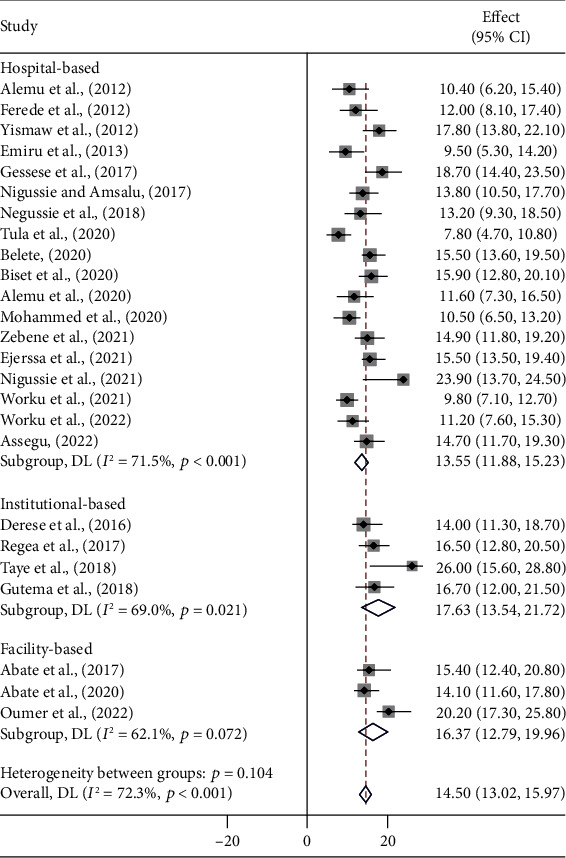
The overall prevalence of UTI in Ethiopia by study nature (participants).

**Figure 6:**
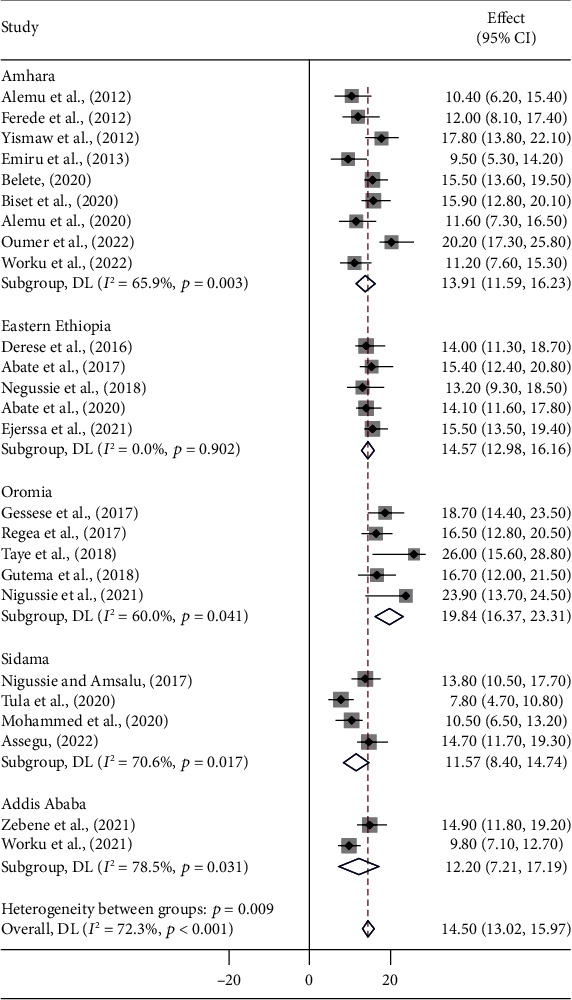
Pooled prevalence of UTIs among patients with DM and PW by region.

**Figure 7:**
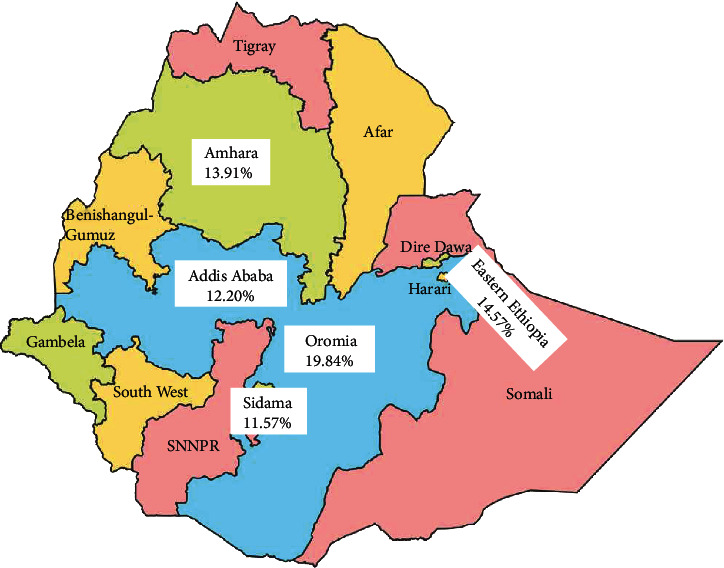
Regional distribution of UTI among diabetes mellitus and pregnant women patients in Ethiopia.

**Figure 8:**
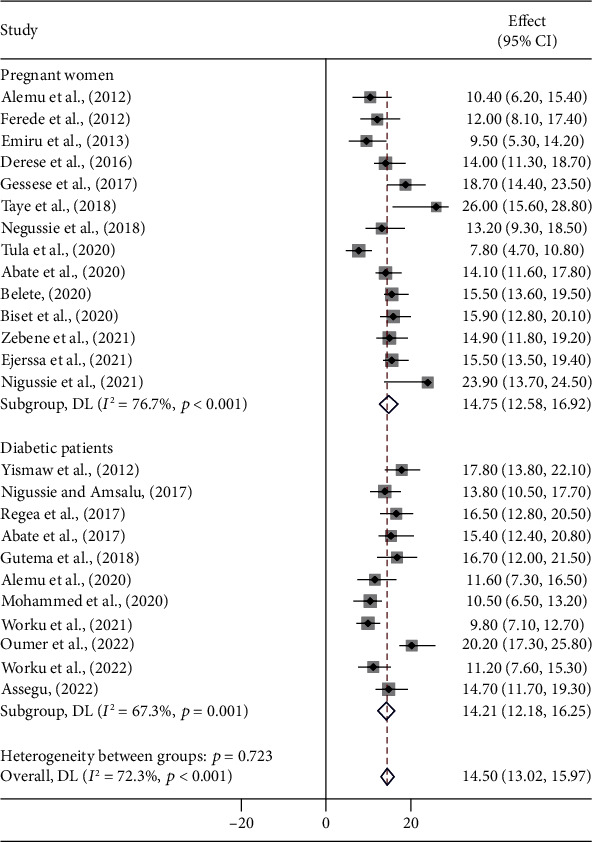
Pooled prevalence of UTI by type of patients (DM and PW).

A meta-regression was performed to identify heterogeneity sources using sample size, year of publication, and study setting as covariates. However, it was indicated that there are effects on heterogeneity between studies, as indicated by a significant *P* value (Table 3, Figures 3–5). Thus, considerable UTI prevalence was reported as 15.64 (95% CI: 12.82, 18.46) in ≤200 sample sizes compared to the counterparts (>200) 14.15 (95% CI: 12.42, 15.87) (Table 3 and Figure 3). The high pooled prevalence of UTI among DM and PW patients was reported from the Oromia region at 19.84% (95% CI: 16.37, 23.31), followed by eastern Ethiopia at 14.57% (95% CI: 12.98, 16.16), Amhara at 13.91% (95% CI: 11.59, 16.23), and Addis Ababa at 12.20% (95% CI: 7.21, 17.19), whereas the low prevalence of UTI was observed in the Sidama region at 11.57% (95% CI: 8.40, 14.74) (Table 3, Figures 6 and 7). The highest pooled prevalence estimate in the study period was recorded between 2018 and 2022: 14.68% (95% CI: 12.66, 16.71), followed by the study period from 2012 to 2017 with a pooled prevalence estimate of 14.30% (95% CI: 12.33, 16.27) (Table 3 and Figure 4). The high pooled prevalence of UTI was reported in pregnant women at 14.75% (95% CI: 12.58, 16.92), followed by diabetic patients at 14.21% (95% CI: 12.18, 16.25) (Table 3 and Figure 8). In the study setting, the highest pooled prevalence estimate was 17.63% (95% CI: 13.54, 21.72) in the institutional-based studies, followed by facility-based studies at 16.37% (95% CI: 12.79, 19.96), and the least at 13.55% (95% CI: 11.88, 15.23) in hospital-based studies (Table 3 and Figure 5).

### 3.4. Factors Associated with DM and PW Patients in Ethiopia

In this systematic review and meta-analysis, we have checked several risk factors for their association with UTI among patients with DM and PW in Ethiopia, such as age (both), sex (DM), education level (both), income level (PW), residence (PW), gestational period (PW), hemoglobin level (PW), history of catheterization (both), previous history of UTI (both), current symptoms of UTI (both), blood glucose level (DM), and type of diabetes (DM). However, only sex (DM), income level (PW), previous history of UTI (both) current symptoms of UTI (both), and history of catheterisation (both) were significantly associated with UTI (S1, S2, S3, S4, and S5).

The association between sex and UTI among patients with DM was analysed in six studies (S1). Female DM patients were 0.88 times more likely to have a UTI than male DM patients (95% CI: 0.11, 1.65, *P* = 0.01). Furthermore, the pooled result of sex was significantly associated with UTI among DM patients (S1).

The pooled results of four studies (S2) showed that income level was significantly associated with UTI among patients. The odds of having a UTI among the income levels were 4.46 times higher for ≤500 ETB than for the parallel (95% CI: -1.19, 10.12, *P* = 0.03).

The association between the previous history of UTI among patients with DM and PW in Ethiopia was calculated from 13 studies (S3). AOR showed that the previous history of UTI among patients with DM and PW was 3.52 (95% CI: 1.96, 5.08, *P* < 0.01) times higher than their counterparts.

The association between the current symptoms and UTI in DM and PW patients was computed from six studies (S4). Symptomatic DM and PW patients were 2.32 (95% CI: 0.57, 4.06, *P* < 0.01) times higher than their asymptomatic counterparts.

The pooled odds result of six studies (S5) showed that the history of catheterization was significantly associated with UTI in both patients. The AOR of having a UTI among DM and PW patients with a history of catheterization was 5.58 times higher than their counterparts (95% CI: 1.35, 9.81, *P* < 0.01).

**Figure 9:**
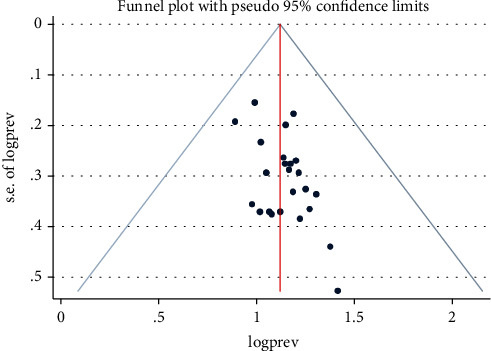
Presentation of the meta-flip chart, an indication of publication bias among studies in Ethiopia from 2012 to 2022.

Twenty-two studies obtained high-quality scores, while three had middle-quality scores when it came to assessing risk bias (Table 1). The most common biases observed were representation and case definition. The pooled prevalence without medium-quality studies was calculated to see how they affected our estimates of pooled prevalence. Our pooled prevalence estimates with and without these studies had confidence intervals that overlapped, indicating that there was no meaningful difference between them (Figure 9). Based on these findings, the majority of the primary study authors met high-quality standards (Figure 9). This gives our findings more credibility.

## 4. Discussion

Urinary tract infections (UTIs) are among the most common infections that affect people of all ages around the world. The most common bacterial infection in pregnancy is the UTI, and it increases the risk of morbidity and mortality in both the mother and the newborn. When bacteriuria strikes during pregnancy, it results in a much higher number of neonates with low birth weight, early birth, and a higher neonatal mortality rate [62]. UTI coinfection among diabetic patients is also becoming a more common cause of morbidity than in normal individuals. Evidence shows that in developing countries, it is highly linked to low-income economies that bear the brunt of the burden due to a lack of resources to combat diseases before they become severe [63–66]. Furthermore, UTIs are common in pregnant women and diabetics due to immune system dysfunction caused by decreased cell responses [67, 68].

The overall pooled prevalence of UTI among DM and PW patients in the present study was 14.50%. This was relatively comparable to the studies conducted in Nekemte (16.5%) [32], Metu (16.7%) [40], and Gondar (17.8%) [25] and outside of Ethiopia, like Kenya (15.8%) [63], Nigeria (17.3%) [64], and Sudan (19.5%) [65]. The result was higher than the reports in Jimma (9.2%) [66], Addis Ababa (9.8%) [57], and Dessie (11.6%) [50] and outside Ethiopia, like Romania (10.7%) [69], Nepal (10.37%) [70], and Uganda (13.3%) [67]. However, the prevalence was lower than the studies reported in Harar (23.0%) [45], Bahir Dar (30.5%) [68], Arba Minch (33.9%) [41], and Shashemene (90.1%) [71] and studies conducted outside Ethiopia, such as in Uganda (31.1%) [72], Kuwait (35%) [73], Malaysia (40.2%) [16], India (49.15%) [74], Pakistan (52.76%) [75], Egypt (52.2%) [76], and Nepal (54.25%) [77]. The magnitude variation could be due to differences in geographical characteristics, study year, host factor, and practises, such as social habits of the community and standards of personal hygiene and health education practises in each country.

In this study, the overall prevalence of UTI among diabetic patients was 14.21% (95% CI: 12.18, 16.25). This was relatively comparable with the studies conducted in Hawassa (14.70%) [61], Addis Ababa (14.9%) [42], and Harar (15.4%) [33] and outside Ethiopia, such as in Uganda (13.3%) [67], Tanzania (13.7%) [78], and Nigeria (17.3%) [79]. However, this finding is not in accordance with the results reported from Bahir Dar (30.5%) [68] and Arba Minch (33.9%) [41] and outside of Ethiopia, such as India (32%) [80], Iraq (49.1%) [81], Nepal (50.7%) [82], Pakistan (51%) [83], and Egypt (52.2%) [76]. The researchers hypothesised that poor circulation, a weakened immune system caused by a decrease in the capacity of white blood cells to fight infections, and poor bladder contractions that result in dysfunctional bladder function were some of the contributing factors that led to an increase in UTI cases in diabetic patients [84–86].

In this study, the overall prevalence of UTI in pregnant women was 14.75% (95% CI: 12.58, 16.92). This was in agreement with similar studies reported in Ethiopia (14.0%) [28], Sudan (14%) [87], Kenya (14.2%) [88], and Tanzania (14.6%) [89]. But it was higher than the studies conducted in Bahir Dar (9.5%) by Demilie et al. [90], in Gondar (10.4%) by Alemu et al. [23], and in Addis Ababa (11.6%) by Assefa et al. [14]. In contrast, it was lower than studies conducted outside Ethiopia in Libya (30%) [91], Iraq (64.6%) [92], and Nigeria (85%) [93]. This difference may be explained by the fact that the environment, social habits of the community, personal hygiene standards, and educational levels in each nation may differ, which could account for the variation in the rate of bacterial UTI aetiologies.

Regarding regions, the highest pooled prevalence estimate of UTI among patients with PW and DM was 19.84% in the Oromia region. This was relatively consistent with the studies conducted in Harar (19.9%) [46] and Gondar (17.8%) [25]. On the other hand, the result was relatively lower than the findings conducted in Arba Minch (33.9%) [41] and Bahir Dar (30.5%) [68] and outside of Ethiopia, in Uganda (31.1%) [72], India (32%) [80], and Kuwait (35%) [73]. While the result was greater than the studies reported from Bahir Dar (9.5%) [26], Addis Ababa (9.8%) [57], Mizan Aman (10.3%) [44], and Dessie (11.6%) [50], this variation could be due to the difference in study year, sample size, patient type (symptomatic or asymptomatic or both), type of identification method used, type of etiological agents studied (bacterial, fungal, protozoan, or all), and study setting (geographical variations).

In this study, the magnitude of UTIs was 0.88 times (95% CI: 0.11, 1.65) more likely to develop in female diabetics than male diabetics, which is in agreement with previously reported studies in Ethiopia [86, 94], Romania [95], Saudi Arabia [96], the United States of America [97], and India [98]. The high prevalence of UTI among the female population might be due to their anatomy and reproductive physiology, such as the decrease of normal vaginal flora (Lactobacilli), the less acidic pH of the vaginal surface, poor hygienic conditions, a short and wide urethra, proximity to the anus, and sexual intercourse, which may force bacteria into the female bladder.

With regard to income level, pregnant women with a family income of less than or equal to 500 ETB were more likely to acquire UTI (AOR: 4.46; 95% CI: -1.19, 10.12) than their counterparts. This result is consistent with the findings conducted in Ethiopia [26], Egypt [99], and Pakistan [100]. This may be demonstrated by the fact that pregnant women with poor socioeconomic status were more likely to be exposed to malnutrition, which had an impact on immunity.

Furthermore, the pooled odds ratio showed that DM and PW patients who had a previous history of UTI were 3.52 times (95% CI: 1.96, 5.08) more likely to acquire UTI than their counterparts. This finding is in parallel with previous findings conducted in the country [31, 86] as well as in other parts of the world [100, 101]. Furthermore, patients with current symptoms of UTI (symptomatic patients) were 2.32 times more likely to develop UTI than asymptomatic patients. The difference could be due to the return of the infection as a result of inadequate therapy and the presence of high sugar concentrations in diabetic urine, which act as a medium for pathogenic bacteria to multiply, or recollection bias.

Regarding catheterization, the odds of a person having a history of catheterization were 5.58 times (95% CI: 1.35, 9.81, *P* < 0.01) more likely to catch a UTI than a person without a previous history of catheterization. This result is in line with the study conducted in Ethiopia [15] and elsewhere [100, 102]. The intrusive procedure of catheterization has the potential to harm the urethral mucosa. Additionally, due to ineffective infection control or inadequate aseptic technique, it may result in the introduction of a bacterial organism that causes haematogenous bacterial dissemination and recurrent UTIs [103].

### 4.1. Limitations of the Study

Small numbers of published papers were collected from the regions involved in this study, and published papers from the Afar, B/Gumuz, SNNPR, and Gambela regions were not included, so the prevalence of UTI and associated risk factors among DM and PW patients may not be fully represented.

## 5. Conclusion and Recommendations

Urinary tract infections and other noncommunicable diseases are becoming more prevalent in developing countries like Ethiopia due to a lack of problem identification, effective treatment, and intervention measures. The overall pooled prevalence of UTI among both DM and PW patients was 14.50%. Being female and having a family income level ≤ 500 ETB had a higher risk of acquiring UTI among DM and PW patients, respectively. Furthermore, patients with a previous history of UTI, catheterization, or symptomatic patients had higher odds of contracting UTI than those who had no previous history of UTI, catheterization, or asymptomatic patients. Increasing the community's knowledge about frequent urine analysis and antenatal care services, blood sugar tests, early diagnosis, and proper medications should be addressed to alleviate the prevalence of UTI in patients with DM and PW.

## Figures and Tables

**Table 1:** List and characteristics of eligible studies included for systematic review from 2012 to 2022.

Authors	Publication year	Region	Study area	Type of patients	Study setting	Study design	Sample taken	Laboratory method used for detection of UTI	Type of UTI (asymptomatic or symptomatic or both)	Etiological agents	Sample size	Case	Prevalence (95% CI)	Quality
Alemu et al.	2012 [23]	Amhara	Gondar	Pregnant women	Hospital based	Cross-sectional	Clean-catch midstream urine	Culture method	Both (13 symptomatic and 27 asymptomatic)	Bacterial	385	40	10.4 (6.2, 15.4)	5
Ferede et al.	2012 [24]	Amhara	Gondar	Pregnant women	Hospital based	Cross-sectional	Clean-catch midstream urine	Culture method	Both (10 symptomatic and 14 asymptomatic)	Bacterial	200	24	12 (8.1, 17.4)	5
Yismaw et al.	2012 [25]	Amhara	Gondar	Diabetic patients	Hospital based	Cross-sectional	Clean-catch midstream urine	Culture method	Both (18 symptomatic and 57 asymptomatic)	Bacterial	422	75	17.8 (13.8, 22.1)	4
Emiru et al.	2013 [26]	Amhara	Bahir Dar	Pregnant women	Hospital based	Cross-sectional	Clean-catch midstream urine	Culture method	Both (7 symptomatic and 28 asymptomatic)	Bacterial	367	35	9.5 (5.3, 14.2)	5
Tadesse et al.	2014 [27]	Sidama	Hawassa	Pregnant women	Hospital based	Cross-sectional	Clean-catch midstream urine	Culture method	Asymptomatic	Bacterial	244	46	18.8 (15.3, 23.4)	5
Girma and Aemiro	2015 [18]	Oromia	Adama	Pregnant women	Hospital based	Cross-sectional	Clean-catch midstream urine	Urine microscopy and culture method	Asymptomatic	Bacterial	367	59	16.1 (13.6, 20.3)	4
Derese et al.	2016 [28]	Eastern	Dire Dawa	Pregnant women	Institutional based	Cross-sectional	Clean-catch midstream urine	Culture method	Both (15 symptomatic and 11 asymptomatic)	Bacterial	186	26	14.0 (11.3, 18.7)	5
Ahmed et al.	2016 [29]	Oromia	Yabello	Pregnant women	Hospital based	Cross-sectional	Clean-catch midstream urine	Urine microscopy, urine dipstic*k* test, and culture method	Both (27 symptomatic and 5 asymptomatic)	Bacterial, yeast, and protozoa	280	31	11.1 (7.2, 15.8)	4
Gessese et al.	2017 [30]	Oromia	Ambo	Pregnant women	Hospital based	Cross-sectional	Clean-catch midstream urine	Culture method	Both (21 symptomatic and 35 asymptomatic)	Bacterial	300	56	18.7 (14.4, 23.5)	6
Nigussie and Amsalu	2017 [31]	Sidama	Hawassa	Diabetic patients	Hospital based	Cross-sectional	Clean-catch midstream urine	Culture method	Both (12 symptomatic and 21 asymptomatic)	Bacterial	240	33	13.8 (10.5, 17.7)	5
Regea et al.	2017 [32]	Oromia	Nekemte	Diabetic patients	Institutional based	Cross-sectional	Clean catch midstream urine	Culture method	Both in symptomatic and asymptomatic	Bacterial	200	33	16.5 (12.8, 20.5)	4
Abate et al.	2017 [33]	Eastern	Harar	Diabetic patients	Facility based	Cross-sectional	Clean catch midstream urine	Culture method	Both (19 symptomatic and 18 asymptomatic)	Bacterial	240	37	15.4 (12.4, 20.8)	5
Habteyohannes et al.	2018 [34]	Amhara	Bahir Dar	Pregnant women	Hospital based	Cross-sectional	Freshly voided midstream urine	Culture method	Asymptomatic	Bacterial	234	27	11.5 (7.6, 16.2)	5
Ali et al.	2018 [35]	Amhara	Dessie	Pregnant women	Hospital based	Cross-sectional	Freshly voided midstream urine	Culture method	Asymptomatic	Bacterial	358	56	15.6 (13.7, 19.6)	5
Taye et al.	2018 [36]	Oromia	Bale	Pregnant women	Institutional based	Cross-sectional	Early morning midstream urine	Culture method	Both (18 symptomatic and 26 asymptomatic)	Bacterial	169	44	26.0 (15.6, 28.8)	5
Negussie et al.	2018 [37]	Eastern	Jigjiga	Pregnant women	Hospital based	Cross-sectional	Clean-catch midstream urine	Culture method	Both (4 symptomatic and 21 asymptomatic)	Bacterial	190	25	13.2 (9.3, 18.5)	5
Tadesse et al.	2018 [38]	Tigray	Adigrat	Pregnant women	Hospital based	Cross-sectional	Freshly voided midstream urine	Culture method	Asymptomatic	Bacterial	259	55	21.2 (17.8, 26.4)	5
Kumera et al.	2018 [39]	Sidama	Hawassa	Diabetic patients	Hospital based	Case-control	Clean catch midstream urine	Culture method	Asymptomatic	Bacterial	100	22	22.0 (17.6, 26.4)	4
Gutema et al.	2018 [40]	Oromia	Metu	Diabetic patients	Institutional based	Cross-sectional	Clean catch midstream urine	Culture method	Both in symptomatic and asymptomatic	Bacterial	233	39	16.7 (12.0, 21.5)	6
Mama et al.	2019 [41]	SNNPR	Arba Minch	Diabetic patients	Facility based	Cross-sectional	Clean catch midstream urine	Culture method	Both (52 symptomatic and 29 asymptomatic)	Bacterial and yeast	239	81	33.9 (28.2, 37.9)	5
Woldemariam et al.	2019 [42]	Addis Ababa	Addis Ababa	Diabetic patients	Hospital based	Cross-sectional	Clean catch midstream urine	Culture method	Both (35 symptomatic and 21 asymptomatic)	Bacterial and yeast	248	56	22.6 (18.4, 27.2)	4
Tula et al.	2020 [43]	Sidama	Hawassa	Pregnant women	Hospital based	Cross-sectional	Clean-catch midstream urine	Culture method	Both (11 symptomatic and 12 asymptomatic	Bacterial	296	23	7.8 (4.7, 10.8)	6
Kumalo and Tadesse	2020 [44]	SNNPR	Mizan Aman	Pregnant women	Hospital based	Cross-sectional	Clean-catch midstream urine	Culture method	Asymptomatic	Bacterial	260	28	10.3 (6.0, 15.0)	5
Abate et al.	2020 [45]	Eastern	Harar	Pregnant women	Facility based	Cross-sectional	Clean-catch midstream urine	Culture method	Both in symptomatic and asymptomatic	Bacterial	638	90	14.1 (11.6, 17.8)	5
Edae et al.	2020 [46]	Eastern	Harar	Pregnant women	Institutional based	Cross-sectional	Clean-catch midstream urine	Culture method	Asymptomatic	Bacterial	283	56	19.9 (16.4, 24.6)	6
Wabe et al.	2020 [47]	Addis Ababa	Addis Ababa	Pregnant women	Hospital based	Cross-sectional	Clean-catch midstream urine	Culture method	Asymptomatic	Bacterial	290	49	16.9 (13.1, 21.5)	6
Belete	2020 [48]	Amhara	Dessie	Pregnant women	Hospital based	Cross-sectional	Freshly voided midstream urine	Culture method	Both (21 symptomatic and 29 asymptomatic)	Bacterial	323	50	15.5 (13.6, 19.5)	5
Biset et al.	2020 [49]	Amhara	Gondar	Pregnant women	Hospital based	Cross-sectional	Clean-catch midstream urine	Culture method	Both (38 symptomatic and 23 asymptomatic)	Bacterial	384	61	15.9 (12.8, 20.1)	6
Alemu et al.	2020 [50]	Amhara	Dessie	Diabetic patients	Hospital based	Cross-sectional	Clean catch midstream urine	Culture method	Both (11 symptomatic and 28 asymptomatic)	Bacterial	336	39	11.6 (7.3, 16.50)	5
Mohammed et al.	2020 [51]	Sidama	Hawassa	Diabetic patients	Hospital based	Cross-sectional	Clean catch midstream urine	Culture method	Both (10 symptomatic and 16 asymptomatic)	Bacterial	247	26	10.5 (6.5, 13.2)	5
Abu et al.	2021 [52]	B/Gumuz	Assosa	Pregnant women	Facility based	Cross-sectional	Freshly voided midstream urine	Culture method	Asymptomatic	Bacterial	283	39	13.8 (10.5, 17.7)	5
Bizuwork et al.	2021 [53]	Addis Ababa	Addis Ababa	Pregnant women	Facility based	Cross-sectional	Clean-catch midstream urine	Culture method	Asymptomatic	Bacterial	281	44	15.7 (13.8, 20.7)	5
Zebene et al.	2021 [54]	Addis Ababa	Addis Ababa	Pregnant women	Hospital based	Cross-sectional	Clean-catch midstream urine	Culture method	Both (14 symptomatic and 49 asymptomatic	Bacterial	424	63	14.9 (11.8, 19.2)	5
Ejerssa et al.	2021 [55]	Eastern	Harar	Pregnant women	Hospital based	Cross-sectional	Clean-catch midstream urine	Culture method	Both (13 symptomatic and 18 asymptomatic	Bacterial	200	31	15.5 (13.5, 19.4)	5
Nigussie et al.	2021 [56]	Oromia	Goba	Pregnant women	Hospital based	Cross-sectional	Freshly voided midstream urine	Culture method	Both (37 symptomatic and 19 asymptomatic	Bacterial	234	56	23.9 (13.7, 24.5)	5
Worku et al.	2021 [57]	Addis Ababa	Addis Ababa	Diabetic patients	Hospital based	Cross-sectional	Freshly voided midstream urine	Culture method	Both (15 symptomatic and 7 asymptomatic	Bacterial	225	22	9.8 (7.10, 12.70)	4
Walelgn et al.	2021 [58]	Amhara	Dessie	Diabetic patients	Hospital based	Cross-sectional	Clean catch midstream urine	Urine microscopy	Both in symptomatic and asymptomatic	Bacterial	359	80	22.3 (18.0, 27.0)	6
Oumer et al.	2022 [59]	Amhara	Kombolcha	Diabetic patients	Facility based	Cross-sectional	Freshly voided midstream urine	Culture method	Both (37 symptomatic and 20 asymptomatic	Bacterial	282	57	20.2 (17.3, 25.8)	5
Worku et al.	2022 [60]	Amhara	Debre Tabor	Diabetic patients	Hospital based	Cross-sectional	Clean catch midstream urine	Culture method	Both (5 symptomatic and 23 asymptomatic	Bacterial	250	28	11.2 (7.6, 15.3)	5
Assegu	2022 [61]	Sidama	Hawassa	Diabetic patients	Hospital based	Cross-sectional	Clean-catch midstream urine	Culture method	Both (17 symptomatic and 27 asymptomatic)	Bacterial	300	44	14.7 (11.7, 19.3)	5

**Table 2:** List and characteristics of eligible studies included for meta-analysis from 2012 to 2022.

Authors	Publication year	Region	Study area	Type of patients	Study setting	Sample size	Case	Prevalence (95% CI)	Quality
Alemu et al.	2012 [23]	Amhara	Gondar	Pregnant women	Hospital based	385	40	10.4 (6.2, 15.4)	5
Ferede et al.	2012 [24]	Amhara	Gondar	Pregnant women	Hospital based	200	24	12 (8.1, 17.4)	5
Yismaw et al.	2012 [25]	Amhara	Gondar	Diabetic patients	Hospital based	422	75	17.8 (13.8, 22.1)	4
Emiru et al.	2013 [26]	Amhara	Bahir Dar	Pregnant women	Hospital based	367	35	9.5 (5.3, 14.2)	5
Derese et al.	2016 [28]	Eastern	Dire Dawa	Pregnant women	Institutional based	186	26	14.0 (11.3, 18.7)	5
Gessese et al.	2017 [30]	Oromia	Ambo	Pregnant women	Hospital based	300	56	18.7 (14.4, 23.5)	6
Nigussie and Amsalu	2017 [31]	Sidama	Hawassa	Diabetic patients	Hospital based	240	33	13.8 (10.5, 17.7)	5
Regea et al.	2017 [32]	Oromia	Nekemte	Diabetic patients	Institutional based	200	33	16.5 (12.8, 20.5)	4
Abate et al.	2017 [33]	Eastern	Harar	Diabetic patients	Facility based	240	37	15.4 (12.4, 20.8)	5
Taye et al.	2018 [36]	Oromia	Bale	Pregnant women	Institutional based	169	44	26.0 (15.6, 28.8)	5
Negussie et al.	2018 [37]	Eastern	Jigjiga	Pregnant women	Hospital based	190	25	13.2 (9.3, 18.5)	5
Gutema et al.	2018 [40]	Oromia	Metu	Diabetic patients	Institutional based	233	39	16.7 (12.0, 21.5)	6
Tula et al.	2020 [43]	Sidama	Hawassa	Pregnant women	Hospital based	296	23	7.8 (4.7, 10.8)	6
Abate et al.	2020 [45]	Eastern	Harar	Pregnant women	Facility based	638	90	14.1 (11.6, 17.8)	5
Belete	2020 [48]	Amhara	Dessie	Pregnant women	Hospital based	323	50	15.5 (13.6, 19.5)	5
Biset et al.	2020 [49]	Amhara	Gondar	Pregnant women	Hospital based	384	61	15.9 (12.8, 20.1)	6
Alemu et al.	2020 [50]	Amhara	Dessie	Diabetic patients	Hospital based	336	39	11.6 (7.3, 16.50)	5
Mohammed et al.	2020 [51]	Sidama	Hawassa	Diabetic patients	Hospital based	247	26	10.5 (6.5, 13.2)	5
Zebene et al.	2021 [54]	Addis Ababa	Addis Ababa	Pregnant women	Hospital based	424	63	14.9 (11.8, 19.2)	5
Ejerssa et al.	2021 [55]	Eastern	Harar	Pregnant women	Hospital based	200	31	15.5 (13.5, 19.4)	5
Nigussie et al.	2021 [56]	Oromia	Goba	Pregnant women	Hospital based	234	56	23.9 (13.7, 24.5)	5
Worku et al.	2021 [57]	Addis Ababa	Addis Ababa	Diabetic patients	Hospital based	225	22	9.8 (7.10, 12.70)	4
Oumer et al.	2022 [59]	Amhara	Kombolcha	Diabetic patients	Facility based	282	57	20.2 (17.3, 25.8)	5
Worku et al.	2022 [60]	Amhara	Debre Tabor	Diabetic patients	Hospital based	250	28	11.2 (7.6, 15.3)	5
Assegu	2022 [61]	Sidama	Hawassa	Diabetic patients	Hospital based	300	44	14.7 (11.7, 19.3)	5

**Table 3:** Prevalence of UTI among diabetes patients and pregnant women in Ethiopia by subgroup analysis.

Variables	Characteristics	Number of studies	Sample size	Prevalence (95% CI)	*I* ^2^, *P* value
Sample size	≤200	6	1145	15.64 (95% CI: 12.82, 18.46)	63.50%, *P* = 0.01
	>200	19	6126	14.15 (95% CI: 12.42, 15.87)	74.30%, *P* < 0.01

Pooled prevalence of UTI by region	Amhara	9	2949	13.91 (95% CI: 11.59, 16.23)	65.90%, *P* < 0.01
	Eastern Ethiopia	5	1454	14.57 (95% CI: 12.98, 16.16)	0.00%, *P* = 0.90
	Oromia	5	1136	19.84 (95% CI: 16.37, 23.31)	60.00%, *P* = 0.04
	Sidama	4	1083	11.57 (95% CI: 8.40, 14.74)	70.60%, *P* = 0.01
	Addis Ababa	2	649	12.20 (95% CI: 7.21, 17.19)	78.50%, *P* < 0.03

Pooled prevalence of UTI by year	2012-2017	9	2540	14. 30 (95% CI: 12.33, 16.27)	50.30%, *P* = 0.04
	2018-2022	16	4731	14.68 (95% CI: 12.66, 16.71)	78.6%, *P* < 0.01

Pooled prevalence of UTI by type of patients	Diabetes patients	11	2975	14.21 (95% CI: 12.18, 16.25)	67.30%, *P* < 0.01
	Pregnant women	14	4296	14.75 (95% CI: 12.58, 16.92)	76.70%, *P* < 0.01

Pooled prevalence of UTI by study setting	Facility based	3	1160	16.37 (95% CI: 12.79, 19.96)	62.10%, *P* = 0.07
	Hospital based	18	5323	13.55 (95% CI: 11.88, 15.23)	71.50%, *P* < 0.01
	Institutional based	4	788	17.63 (95% CI: 13.54, 21.72)	69.00%, *P* = 0.02

*Overall*		*25*	*7271*	*14.50 (95% CI: 13.02, 15.97)*	*72.30%,P*<*0.01*

## Data Availability

The data generated and analysed during this study are included in this article.

## Abbreviations

AOR: Adjusted odds ratio
CI: Confidence interval
DM: Diabetes mellitus
ETB: Ethiopian birr
GRADE: Grading of recommendations assessment, development, and evaluation
PRISMA: Preferred reporting items for systematic reviews and meta-analyses
PW: Pregnant women
SNNPR: South nations nationality people region
STATA: Statistical software for data science
UTI: Urinary tract infection.

## Supplementary Materials

Supplementary Materials Supplementary Materials S1: sex as an associated risk factor for UTI among patients with DM. S2: income level as an associated risk factor for UTI among PW patients. S3: previous history of UTI as an associated risk factor for UTI among DM and PW patients. S4: current symptoms of UTI as an associated risk factor for UTI among DM and PW patients. S5: history of catheterization as an associated risk factor for UTI among DM and PW patients.
