# Supplementary figures and images for: Magnitude, Associated Risk Factors, and Trend Comparisons of Urinary Tract Infection among Pregnant Women and Diabetic Patients: A Systematic Review and Meta-Analysis
Source: J Pregnancy. 2023 Jul 28;2023:8365867. doi: 10.1155/2023/8365867 (PMC10403334; doi:10.1155/2023/8365867)

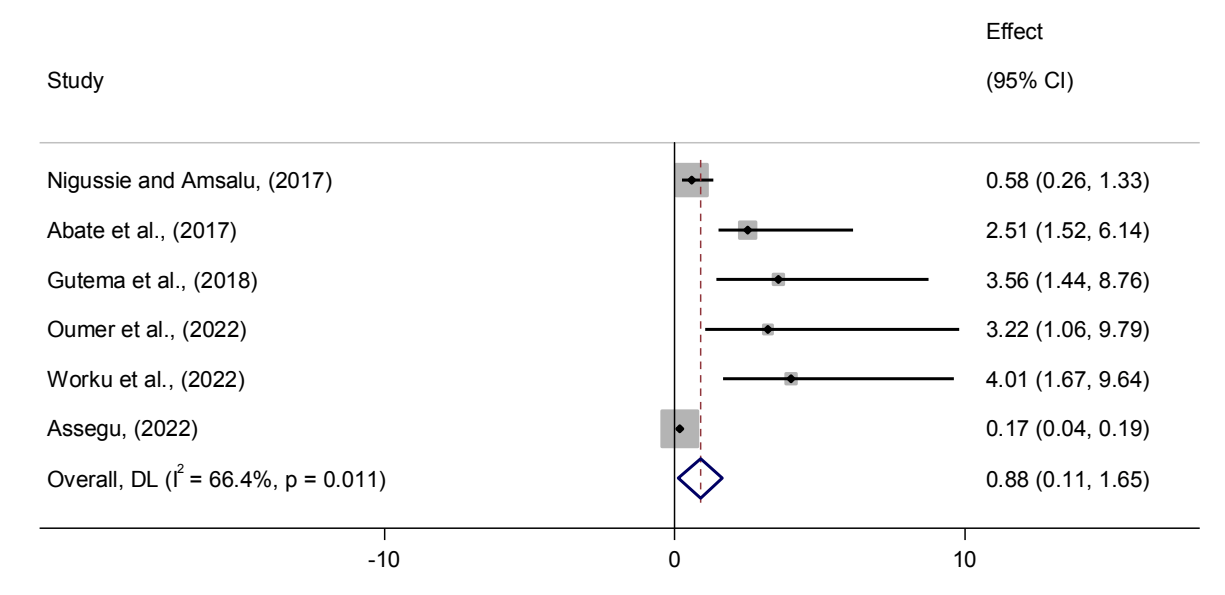

**S1:** Sex as an associated risk factor for UTI among patients with DM

Supplement: Supplementary Materials — S1: sex as an associated risk factor for UTI among patients with DM. S2: income level as an associated risk factor for UTI among PW patients. S3: previous history of UTI as an associated risk factor for UTI among DM and PW patients. S4: current symptoms of UTI as an associated risk factor for UTI among DM and PW patients. S5: history of catheterization as an associated risk factor for UTI among DM and PW patients. [file 8365867.f1.zip › S1.pdf]

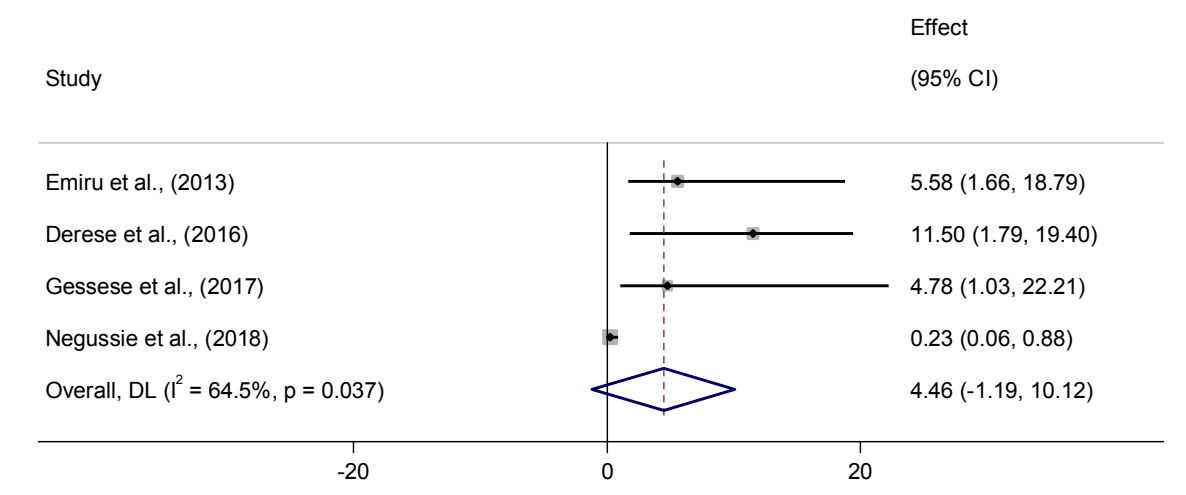

**S2:** Income level as an associated risk factor for UTI among PW patients

Supplement: Supplementary Materials — S1: sex as an associated risk factor for UTI among patients with DM. S2: income level as an associated risk factor for UTI among PW patients. S3: previous history of UTI as an associated risk factor for UTI among DM and PW patients. S4: current symptoms of UTI as an associated risk factor for UTI among DM and PW patients. S5: history of catheterization as an associated risk factor for UTI among DM and PW patients. [file 8365867.f1.zip › S2.pdf]

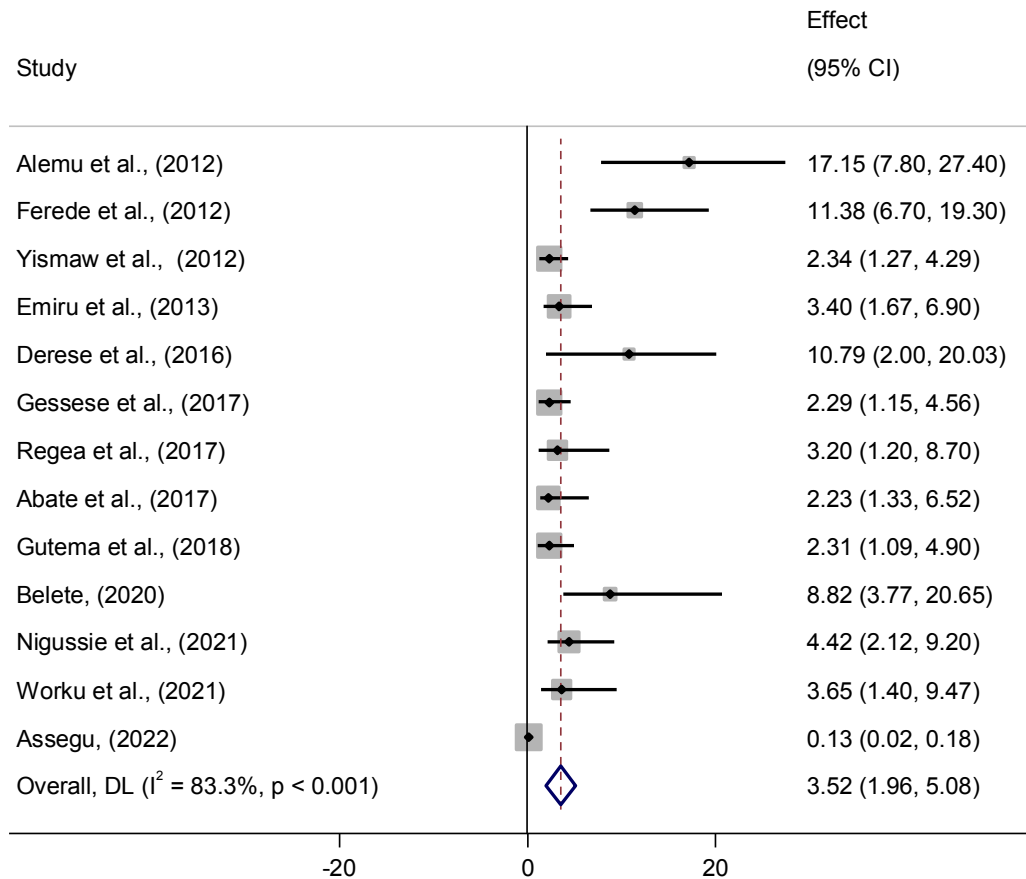

**S3:** Previous history of UTI as an associated risk factor for UTI among DM and PW patients

Supplement: Supplementary Materials — S1: sex as an associated risk factor for UTI among patients with DM. S2: income level as an associated risk factor for UTI among PW patients. S3: previous history of UTI as an associated risk factor for UTI among DM and PW patients. S4: current symptoms of UTI as an associated risk factor for UTI among DM and PW patients. S5: history of catheterization as an associated risk factor for UTI among DM and PW patients. [file 8365867.f1.zip › S3.pdf]

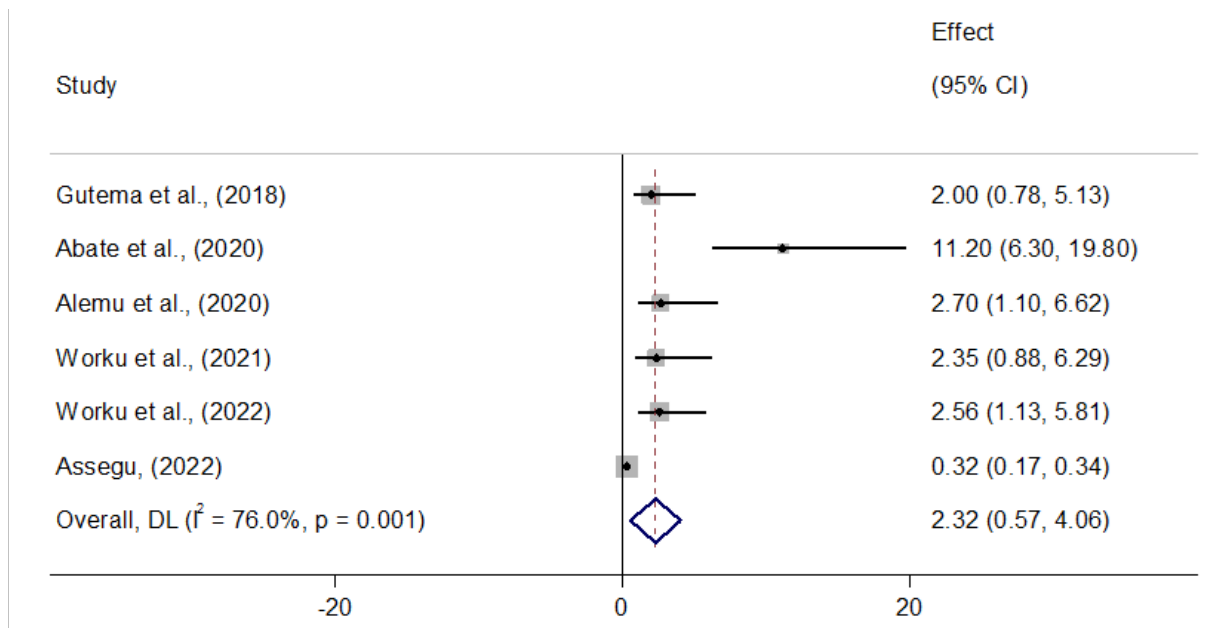

**S4:** Current symptoms of UTI as an associated risk factor for UTI among DM and PW patients.

Supplement: Supplementary Materials — S1: sex as an associated risk factor for UTI among patients with DM. S2: income level as an associated risk factor for UTI among PW patients. S3: previous history of UTI as an associated risk factor for UTI among DM and PW patients. S4: current symptoms of UTI as an associated risk factor for UTI among DM and PW patients. S5: history of catheterization as an associated risk factor for UTI among DM and PW patients. [file 8365867.f1.zip › S4.pdf]

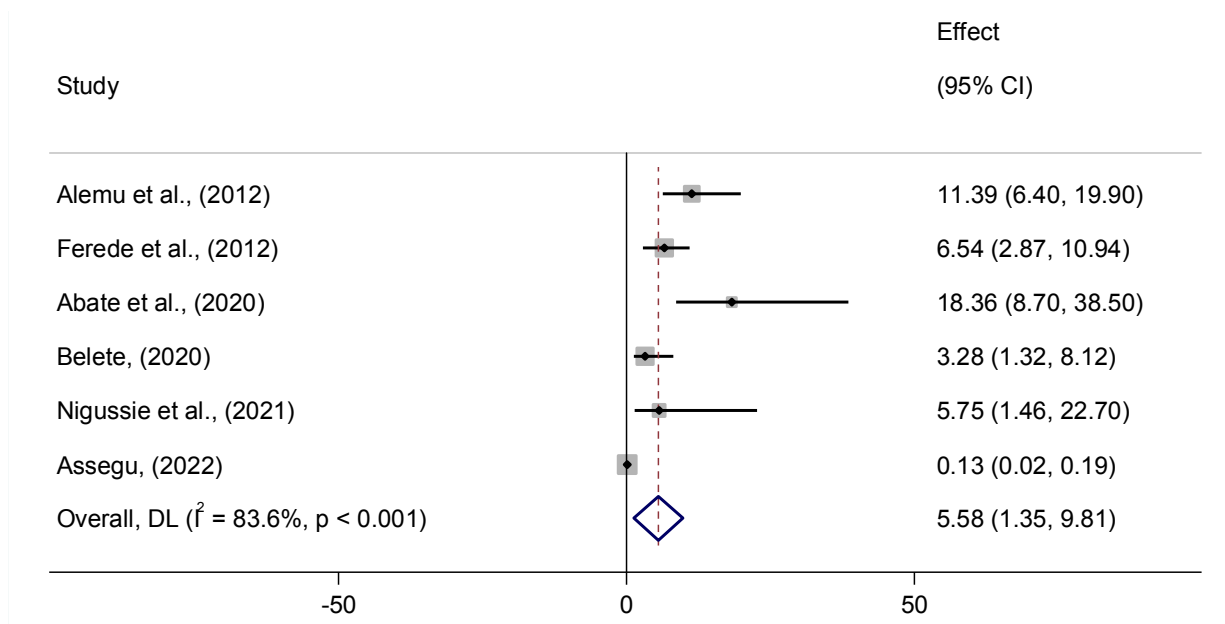

**S5:** History of catheterization as an associated risk factor for UTI among DM and PW patients.

Supplement: Supplementary Materials — S1: sex as an associated risk factor for UTI among patients with DM. S2: income level as an associated risk factor for UTI among PW patients. S3: previous history of UTI as an associated risk factor for UTI among DM and PW patients. S4: current symptoms of UTI as an associated risk factor for UTI among DM and PW patients. S5: history of catheterization as an associated risk factor for UTI among DM and PW patients. [file 8365867.f1.zip › S5.pdf]
